# *HLA-DRB1* among patients with Vogt-Koyanagi-Harada disease in Saudi Arabia

**Published:** 2009-09-12

**Authors:** Alia Iqniebi, Ameera Gaafar, Atia Sheereen, Abdullah Al-Suliman, Gamal Mohamed, Khaled Al-Hussein, Khalid F. Tabbara

**Affiliations:** 1 Histocompatibility & Immunogenetics Research Unit, Stem Cell Therapy Program, King Faisal Specialist Hospital and Research Centre, Riyadh, Saudi Arabia; 2 Disease Control Strategy Group, Liverpool School of Tropical Medicine, Liverpool, UK; 3 The Eye Center and The Eye Foundation for Research in Ophthalmology, Riyadh, Saudi Arabia; 4 Division of Ophthalmology, Department of Surgery, King Faisal Specialist Hospital and Research Centre, Riyadh, Saudi Arabia; 5 The Wilmer Ophthalmological Institute of The Johns Hopkins University School of Medicine, Baltimore, MD

## Abstract

**Purpose:**

Vogt-Koyanagi-Harada (VKH) disease is an immune-mediated disorder with autoimmune insult directed against antigens associated with melanocytes. The genetic predisposition among VKH has not been explored in Saudi Arabia. So, the purpose of this study was to investigate the association of human leukocyte antigen (*HLA*)-*DRB1* alleles to VKH patients and to clarify the molecular genetic mechanism underlying the susceptibility or resistance to VKH disease.

**Methods:**

Genomic DNA from a total of 30 patients with VKH and 29 control subjects was extracted from peripheral blood, and HLA-DRB1 alleles were typed by polymerase chain reaction and sequence based typing (SBT).

**Results:**

We found a statistically significant difference in the prevalence of HLA-DRB1 *0405 between the VKH patients and control subjects (p<0.05). Eleven out of thirty (36.6%) patients with VKH had positive HLA-DRB1 *0405 compared to two out of twenty-nine (6.9%) control subjects. However, there were no statistically significant differences in the HLA-DRB1 alleles *01, *0101, *0102, *0301, *04, *0403, *0404, *0701, *1001, *1101, *1112, *1301, *1302, *1303, *1501, and *1502 between the VKH patients and controls.

**Conclusions:**

Patients with VKH had significantly greater incidence of HLA-DRB1 *0405 when compared to age and sex-matched controls. Consequently, this finding suggests that HLA-DRB1 *0405 allele might play a role in the pathogenesis of VKH disease.

## Introduction

Vogt-Koyanagi-Harada (VKH) disease is a multisystem autoimmune disorder mediated by Th1 lymphocytes reacting against antigens associated with the melanocytes such as tyrosinase and related proteins [1-5]. The disease afflicts the uvea in the eye leading to chronic inflammation and may lead to loss of vision [6]. The disease occurs in stages with early involvement of the meninges followed by auditory, ocular, and integumentary structures. During the early manifestation of the disease, patients may develop malaise, fever, headache, nausea, neck stiffness, and tinnitus. Following an acute onset, the disease becomes chronic and progressive with bilateral choroiditis and panuveitis associated with nummular chorioretinal lesions or exudative retinal detachment. Patients may present with bilateral diffuse granulomatous uveitis. No cutaneous manifestations are noted during the acute phase of the disease, but alopecia, vitiligo, and poliosis may subsequently appear weeks or months after the acute onset of the disease. Tinnitus and dysacusia may occur during the initial phases of the disorder. Thus far, there are no serodiagnostic tests for this disease. and the diagnosis remains clinical. The disease presents with bilateral ocular involvement alone (probable) VKH or bilateral ocular involvement and central nervous system (CNS) and integumentary involvement (complete) VKH or with either CNS or integumentary involvement (incomplete) VKH [7].

In VKH patients, there is no history of penetrating ocular trauma and no clinical or laboratory evidence suggestive of other ocular disorders. In the complete form during acute onset, there is evidence of choroiditis with a focal area of subretinal fluid or bullous exudative retinal detachment. There is diffuse choroidal thickening and multifocal chorioretinal infiltrates. In the late manifestation of the disease, patients may develop sunset glow fundus, alopecia, poliosis, and vitiligo. In the incomplete form of VKH, patients show evidence of bilateral ocular involvement. There is an acute phase of malaise, fever, headache, nausea, and neck stiffness or alopecia, poliosis, or vitiligo. In the probable form of VKH, it is bilateral ocular involvement with no association of integumentary findings or neurologic findings. VKH appears to occur commonly among communities with dark pigment such as Native Americans, Middle Easterners, Asians, and Indians but not in blacks of sub-Saharan African descent. The disease is uncommon among Caucasians [7]. Increased risk among those with certain HLA genotypes showing strong association with human leukocyte antigen (*HLA*)-*DRB1*  *0405 and *HLA-DRB1* *0401 have been reported in several populations [8-12]. In Saudi Arabia, VKH is a common cause of uveitis, yet there has been no previous study on the genetic predisposition among patients with VKH disease [6]. Thus, we intended to explore and analyze the frequency and the association of HLA-DRB1 alleles among patients with VKH in Saudi Arabia and to investigate its potential role in the disease manifestation.

## Methods

### Study population

Thirty patients (12 male and 18 female) with confirmed VKH as defined by the revised diagnostic criteria in the report of the International Committee on Nomenclature [7] were recruited for this study. Patients were divided into two groups, (1) complete VKH disease and (2) incomplete VKH disease, to find out whether there is any genetic difference in patients with different clinical manifestations [7]. Patients with probable VKH were not included. Twenty**-**nine age- and sex-matched healthy volunteers who attended the Blood Bank at King Faisal Specialist Hospital and Research Centre (KFSHRC) (Riyadh, Saudi Arabia) were included as the control. This study was approved by the King Faisal Specialist Hospital and Research Centre Institutional Review Board and maintained the strict adherence to the Declaration of Helsinki for research involving human subjects. Written informed consent was obtained from all the participants before study enrollment.

### Diagnostic criteria

Patients with complete or incomplete VKH were investigated in this study. The diagnosis of complete VKH disease was made as previously described [7]. The diagnosis of complete VKH when the following five criteria: (1) no history of penetrating injury or surgery; (2) no clinical or laboratory evidence suggestive of other ocular diseases; (3) bilateral ocular involvement consisting of anterior uveitis and diffuse or multifocal choroiditis with or without evidence of a retinal detachment. Late manifestations or ocular findings consist of areas of retinochoroidal depigmentations, nummular chorioretinal depigmented scar, retinal epithelial clumping, and peripapillary chorioretinal atrophy with or without chronic anterior uveitis, (4) neurologic/auditory findings include meningismus, malaise, fever, headache, stiffness of the back or neck, tinnitus or cerebrospinal fluid (CSF) pleocytosis, and (5) integumentary findings of alopecia, poliosis, and vitiligo.

The diagnosis of incomplete VKH were made by the following criteria: (1) no history of penetrating ocular injury, (2) no clinical or laboratory evidence suggestive of other ocular disease, (3) bilateral ocular involvement, and (4) neurologic auditory findings and/or cutaneous findings such as vitiligo and poliosis [7].

### Extraction of genomic DNA

Genomic DNA was isolated from the peripheral blood using the Puregene ^TM^ extraction Kit (Gentra Inc., Foster City, CA) following the manufacturer’s recommendation. The extracted DNA was stored at –80 °C for long-term usage. The DNA purity and concentration was determined using a NanoDrop spectrophotometer (Thermo Scientific, Wilmington, DE).

### Determination of HLA-DRB1 allele

The determination of the HLA-DRB1 alleles was performed by using the Allele SEQR HLA Sequencing kit with Heterozygous ambiguity resolving primers (AlleleSEQR HLA Sequencing kit+ HARPs; Atria Genetics, Inc. South San Francisco, CA). The principle of this test relies on two key technologies that are described below.

#### PCR amplification of the *HLA-DRB1* gene

The genes of interest were amplified using 8 µl of gene-specific polymerase chain reaction (PCR) pre-mixes (gene specific oligonucleotide primer mixes in a Tris-based PCR buffer containing dNTPs and MgCl_2_) and 0.1 µl AmpliTaq Gold (Applied Biosystems Inc. Foster City, CA). A total of 40 ng of genomic DNA was added, and the volume was finally adjusted to 10 µl with sterile water. Amplification of the gene was done on PCR thermal cycler (MJ Research,GMI, Ramsay, Minnesota). PCR was initiated with an initial denaturation at 95 °C for 10 min followed by amplification of 36 cycles at 96 °C for 20 s, 60 °C for 30 s , and finally 72 °C for 3 min. This was followed by cleaning of the amplified product using ExoSAP-IT (Exonuclease I and Shrimp Alkaline Phosphatase, together in a specially formulated buffer) from Usb Corporation (Cleveland, OH) and was stored for future use.

#### Sequencing of *HLA-DRB1* alleles

The amplified product serves as a DNA sequencing template in the reaction. Eight microliters of sequencing mix was dispensed in separate reaction tubes with 2 µl of the ExoSAP-treated PCR product. The thermal condition for the sequencing reaction was as follows: 25 cycles for 20 s at 96 °C, 30 s at 50 °C, and 2 min at 60 °C. Finally, the sequence reaction was purified using DyeEx 2.0 spin kit (Qiagen Inc. Valencia, CA) and then suspended with Hi Di Formamide (Applied Biosystems, Foster city,CA) to run on the 3130xl Genetic Analyzer (Applied Biosystems).

### Statistical analysis

This is a case-control study in which the frequency of *HLA-DRB1* alleles in Saudi patients with VKH disease was compared with the frequency of *HLA-DRB1* alleles in a similar group of Saudis without the VKH disease (controls). The risk of the disease was estimated by the odds ratio and calculation of the 95% confidence interval of the odds ratio. A p-value of less than 0.05 was considered significant.

## Results

A total of 30 patients with VKH from the Eye Center and the Eye Foundation for Research in Ophthalmology (Riyadh, Saudi Arabia) were recruited during the period 2007-2008. There were 15 patients with complete VKH and 15 patients with incomplete VKH. A total of 29 healthy volunteer subjects were included. The healthy volunteers were not related to patients with VKH or to each other and were of the same ethnic origin.

Table 1 summarizes the gene frequencies of *HLA-DRB1* alleles for VKH patients and the control group. No significant differences were observed in *HLA-DRB1* frequencies between the patients with VKH and control group. However, when the *HLA-DRB1* *04 were subtyped, the *HLA-DRB1* *0405 allele was found to be positively associated with VKH in the Saudi population (p=0.01). The frequency of *HLA-DRB1* *0401 and *HLA- DRB1* *0410 were measured to be very low in the normal Saudi population (unpublished). Accordingly, these alleles were not detected in our study population (VKH patients and matched controls). On the other hand, the *HLA-DRB1* *0301 and *0403 alleles were found to be higher in the control group than what have been observed in the patients with VKH despite achieving statistically insignificant association, indicating a possible protective role of these two alleles against the disease (Table 1). To assess the significance of the association of *HLA-DRB1* *0405 alleles and the VKH disease, the association of *HLA-DRB1* *0405 with complete VKH and incomplete VKH was analyzed. Five out of fifteen cases of complete VKH and 6 out of 15 patients with incomplete VKH had positive *HLA-DRB1* *0405 with no statistically significant difference (data not shown). The frequency of *HLA-DBR1* *0701 allele was found to be the highest among all the alleles of *HLA-DRB1* in both VKH patients and controls. On the other hand, no statistically significant differences were detected in the *HLA-DRB1* alleles *01, *0101, *0102, *0301, *04, *0403, *0404, *0701, *1001, *1101, *1112, *1301, *1302, *1303, *1501, and *1502 (Table 1).

**Table 1:** Allele frequencies of the HLA- DRB1 in VKH patients and in controls.

***HLA-DRB1* alleles**	**Control (n=29) alleles (n=58)**		**Patient (n=30) alleles (n=60)**		**OD**	**CI**	**p**
	**Number**	**%**	**Number**	**%**			
*01							
*0101	2	3.5	1	1.7	0.475	0.04–5.24	0.54
*0102	2	3.5	3	5	1.47	0.24–9.2	0.65
*03							
*0301	8	13.8	5	8.3	0.57	0.17–1.9	0.34
*0302	1	1.7	1	1.7	0.97	0.06–15.8	0.98
*04							
*0403	5	8.6	1	1.7	0.18	0.02–1.6	0.09
*0404	1	1.7	3	5	3	0.3–29.7	0.33
*0405	**2**	3.5	**11**	18.3	6.3	1.3–29.7	**0.01**
*0406	1	1.7	1	1.7	0.97	0.06–15.8	0.98
*0408	1	1.7	1	1.7	0.97	0.06–15.8	0.98
*0417	1	1.7	2	3.3	1.97	0.17–22.3	0.6
*0437	1	1.7	1	1.7	0.97	0.06–15.8	0.98
*0701	18	31.3	17	28.3	0.88	0.4–1.9	0.76
*0804	1	1.7	1	1.7	0.97	0.06–15.8	0.98
*1001	1	1.7	2	3.3	1.97	0.17–22.3	0.6
*11							
*1101	2	3.5	1	1.7	0.475	0.04–5.24	0.54
*1112	2	3.5	1	1.7	0.475	0.04–5.24	0.54
*1202	1	1.7	2	3.3	1.97	0.17–22.3	0.6
*13							
*1301	1	1.7	1	1.7	0.97	0.06–15.8	0.98
*1302	5	8.6	3	5	0.56	0.12–2.4	0.4
*1303	1	1.7	1	1.7	0.97	0.06–15.8	0.98
*1352	1	1.7	1	1.7	0.97	0.06–15.8	0.98
*15							
*1501	4	6.9	4	6.7	0.96	0.23–4.1	0.96
*1502	1	1.7	1	1.7	0.97	0.06–15.8	0.98
*16							
*1602	1	1.7	2	3.3	1.97	0.17–22.3	0.6
*1605	1	1.7	1	1.7	0.97	0.06–15.8	0.98

Table 1 shows the *HLA-DRB1* allele frequencies in VKH patients and controls. The bold numbers in the table indicates allele *0405, showing a significant p value. Abbreviations in the table are, OD; Odds Ratio, CI; Confidence Interval.

## Discussion

Association of the *HLA-DRB1* *0405 allele and VKH disease has already been reported in the other races [8,11,12]. However, this is the first report studying the genetic predisposition of a very important autoimmune disease, Vogt-Koyanagi-Harada Disease (VKH), in Saudi Arabia. We performed complete *HLA-DRB1* genotyping and found that *HLA-DRB1* *0405 was significantly associated with VKH disease, and no such association was noted with *HLA-DRB1* *0404. The association between class I antigen and VKH disease may differ among various ethnic groups. Among Koreans, *HLA-DRB1* *04 was positive in 17 (94.4%) out of 18 patients [11]. Although the clinical manifestations of VKH are well outlined [7], the exact etiology of this condition remains to be elucidated. It has been suggested the mechanism could be a T-lymphocyte-mediated autoimmune process directed against an unidentified antigen or a group of antigens associated with the melanocytes [1,4,5]. Although the mechanism that triggers this autoimmune attack against melanocytes is unknown, sensitization to melanocytes has been proposed [5]. While the exact target antigen has not been identified, several candidates were proposed. This includes tyrosinase or tyrosinase-related proteins and a 75 kDa protein obtained from cultured human melanoma cells [13]. The etiology of VKH syndrome is not certain, but the clinical history of VKH syndrome mimics an influenza attack, which implies a viral or post infectious origin. An Epstein-Barr virus reactivation in this disease has been suggested [14]. Although a viral cause has been projected, no virus has been isolated or cultured from patients with VKH syndrome. Morris and Schlaegel [15] found virus-like inclusion bodies in the subretinal fluid of a patient with the VKH syndrome. An association between *HLA-DRB1* *0405 and VKH has been noted among patients from Japan, Brazil, Korea, and Mexico [8,9,11,12,16]. Therefore, a genetically determined susceptibility to the triggering event for the VKH disease has been suggested [5,17]. The data presented here confirmed an association of *HLA-DRB1* *0405 with the VKH patients in Saudi Arabia. This relationship was similar to those reported from Japan and Brazil [8,12]. Out of the *HLA-DRB1* *04 subtypes, *HLA-DRB1* *0405 had the strongest association with the VKH disease [11]. The association of *HLA-DRB1* *0405 among Brazilian patients with VKH may reflect a genetic pool inherited from Japanese descendants. Kim and associates found that *HLA-DRB1* *0405 was greatly increased in patients with VKH syndrome among Koreans and might have an important role in the development of the clinical course of VKH [11]. On the other hand, *HLA-DRB1* alleles such as *0705, *1001, *1101, *1112,*1301, *1302, *1303, *1501, and *1502 showed no statistically significant differences between VKH patients and controls. Genetic predisposition and environmental triggers may play a role in the pathogenesis of VKH in Saudi Arabia. Alternatively, the diagnostic criteria of the VKH disease were revised recently in a cohort of VKH Brazilian patients wherein no association was found between disease categories, the presence of *HLA-DRB1* *0405, and the clinical parameters [16]. We think that differences in the genetic background in different geographical areas might contribute to these discrepancies and that more studies need to be done before the previous statement can be generalized. In Korean patients with VKH disease, *HLA-DQA1* *0301 was less frequently detected than in normal control subjects whereas the frequency of *HLA-DQA1* *0302 was increased [11]. These findings suggest that gene association with VKH disease in the HLA region is located between the *HLA- DRB1* locus and the *HLA-DQB1* locus. Shindo and others [8] have reported the presence of *HLA-DRB1* *0405 and/or *HLA-DRB1* *0410 in the VKH patients. Moreover, they reported that the VKH patients who did not have *HLA-DRB1* *0405 possess *HLA-DRB1* *0410 alleles, suggesting that the susceptibility to VKH disease was determined by the presence of the shared functional epitope common to *HLA-DRB1* *0405 and *HLA-DRB1* *0410 [8,18]. But, these alleles were not detected in our study population in either the VKH patients or their matched controls as the frequency of *HLA- DRB1* *0401 and *HLA-DRB1* *0410 were measured to be very low in the normal Saudi population (unpublished). To conclude, patients with VKH had a higher incidence of *HLA-DRB1* *0405, indicating that the *HLA-DRB1* *0405 allele might play a role in the pathogenesis of VKH disease.

## References

[1] Sugita S Sagawa K Mochizuki M Shichijo S Itoh K Melanocyte lysis by cytotoxic T lymphocytes recognizing the MART-1 melanoma antigen in HLA-A2 patients with Vogt-Koyanagi-Harada disease. Int Immunol 1996 8 799 803 8671669 10.1093/intimm/8.5.799

[2] Maezawa N Yano A Taniguchi M Kojima S The role of cytotoxic T lymphocytes in the pathogenesis of Vogt-Koyanagi-Harada disease. Ophthalmologica 1982 185 179 86 6982444 10.1159/000309240

[3] Yokoyama MM Matsui Y Yamashiroya HM O'Donnell MJ Tseng CH Snyder DA Tessler HH Crispen RG Zimjewski CM Humoral and cellular immunity studies in patients with Vogt-Koyanagi-Harada syndrome and pars planitis. Invest Ophthalmol Vis Sci 1981 20 364 70 7203881

[4] Norose K Yano A Melanoma specific Th1 cytotoxic T lymphocyte lines in Vogt-Koyanagi-Harada disease. Br J Ophthalmol 1996 80 1002 8 8976730 10.1136/bjo.80.11.1002 PMC505680

[5] Damico FM Cunha-Neto E Goldberg AC Iwai LK Marin ML Hammer J Kalil J Yamamoto JH T-cell recognition and cytokine profile induced by melanocyte epitopes in patients with HLA-DRB1*0405-positive and -negative Vogt-Koyanagi-Harada uveitis. Invest Ophthalmol Vis Sci 2005 46 2465 71 15980237 10.1167/iovs.04-1273

[6] Tabbara KF Chavis PS Freeman WR Vogt-Koyanagi-Harada syndrome in children compared to adults. Acta Ophthalmol Scand 1998 76 723 6 9881561 10.1034/j.1600-0420.1998.760619.x

[7] Read RW Holland GN Rao NA Tabbara KF Ohno S Arellanes-Garcia L Pivetti-Pezzi P Tessler HH Usui M Revised diagnostic criteria for Vogt-Koyanagi-Harada disease: report of an international committee on nomenclature. Am J Ophthalmol 2001 131 647 52 11336942 10.1016/s0002-9394(01)00925-4

[8] Shindo Y Inoko H Yamamoto T Ohno S HLA-DRB1 typing of Vogt-Koyanagi-Harada's disease by PCR-RFLP and the strong association with DRB1*0405 and DRB1*0410. Br J Ophthalmol 1994 78 223 6 7908535 10.1136/bjo.78.3.223 PMC504742

[9] Levinson RD See RF Rajalingam R Reed EF Park MS Rao NA Holland GN HLA-DRB1 and -DQB1 alleles in mestizo patients with Vogt-Koyanagi-Harada's disease in Southern California. Hum Immunol 2004 65 1477 82 15603876 10.1016/j.humimm.2004.07.236

[10] Gupta A Kamal S Gupta V Bambery P Kaura B HLA typing in Vogt-Koyanagi-Harada syndrome in North Indian patients. Ocul Immunol Inflamm 2007 15 89 97 17558833 10.1080/09273940601186727

[11] Kim MH Seong MC Kwak NH Yoo JS Huh W Kim TG Han H Association of HLA with Vogt-Koyanagi-Harada syndrome in Koreans. Am J Ophthalmol 2000 129 173 7 10682969 10.1016/s0002-9394(99)00434-1

[12] Goldberg AC Yamamoto JH Chiarella JM Marin ML Sibinelli M Neufeld R Hirata CE Olivalves E Kalil J HLA-DRB1*0405 is the predominant allele in Brazilian patients with Vogt-Koyanagi-Harada disease. Hum Immunol 1998 59 183 8 9548078 10.1016/s0198-8859(97)00265-6

[13] Otani S Sakurai T Yamamoto K Fujita T Matsuzaki Y Goto Y Ando Y Suzuki S Usui M Takeuchi M Kawakami Y Frequent immune response to a melanocyte specific protein KU-MEL-1 in patients with Vogt-Koyanagi-Harada disease. Br J Ophthalmol 2006 90 773 7 16481377 10.1136/bjo.2005.086520 PMC1860214

[14] Sunakawa M Okinami S Epstein-Barr virus-related antibody pattern in uveitis. Jpn J Ophthalmol 1985 29 423 8 3007826

[15] Schlaegel TF Jr Morris WR Viruslike Inclusion Bodies in Subretinal Fluid in Uveo-Encephalitis. Am J Ophthalmol 1964 58 940 5 14233716 10.1016/0002-9394(64)90002-9

[16] da Silva FT Damico FM Marin ML Goldberg AC Hirata CE Takiuti PH Olivalves E Yamamoto JH Revised diagnostic criteria for vogt-koyanagi-harada disease: considerations on the different disease categories. Am J Ophthalmol 2009 147 339 345.e5 18992868 10.1016/j.ajo.2008.08.034

[17] Prasad PS Levinson RD In silico prediction of binding of putative antigenic peptides to HLA-DRB1 alleles in Vogt-Koyanagi-Harada disease. Clin Immunol 2005 116 143 8 15927531 10.1016/j.clim.2005.04.012

[18] Shindo Y Ohno S Yamamoto T Nakamura S Inoko H Complete association of the HLA-DRB1*04 and -DQB1*04 alleles with Vogt-Koyanagi-Harada's disease. Hum Immunol 1994 39 169 76 8026985 10.1016/0198-8859(94)90257-7

